# Family history of malignant neoplasm and its relation with clinicopathologic features of gastric cancer patients

**DOI:** 10.1186/1477-7819-11-201

**Published:** 2013-08-16

**Authors:** Junxiu Yu, Bo Fu, Qi Zhao

**Affiliations:** 1 Department of Gastrointestinal Surgery, Liaocheng People’s Hospital and Liaocheng Clinical School of Taishan Medical University, 67 West Dongchang Road, Liaocheng, Shandong Province 252000, China; 2 Department of Gastroenterology, Liaocheng People’s Hospital and Liaocheng Clinical School of Taishan Medical University, 67 West Dongchang Road, Liaocheng, Shandong Province 252000, China

**Keywords:** Gastric cancer, Family history, Malignant neoplasm, Sex, Age, Histological subtypes

## Abstract

**Background:**

Few studies to date have evaluated gastric cancer(GC)-related malignant neoplasm family history (MN-FH), and their findings have been largely inconsistent. The aim of this study is to evaluate the prevalence of MN-FH and its relation to the clinicopathologic features of GC.

**Methods:**

A total of 104 hospitalized patients with primary gastric adenocarcinoma was prospectively analyzed from 2008 to 2009. Positive MN-FH was defined as MN-affected first- and second-degree relatives of the current GC cases. The relation between prevalence of positive MN-FH and clinicopathologic features in the current GC patients was assessed using the Chi-square test with Cramer’s V coefficient.

**Results:**

Thirty-seven (35.6%) of the GC patients had positive MN-FH, with 42 associated tumors in first- and second-degree relatives. Twenty-six (61.9%) of the associated tumors were located in the digestive system, including the esophagus (26.2%), stomach (23.8%), liver (9.5%) and colon (2.4%). Lung cancers were the most prevalent non-digestive system-associated tumors (9.5%). Correlation analysis revealed no significant relations with prevalence of MN-FH and any of the clinicopathologic features (all, *P *> 0.05), including sex (V = 0.044), age (V = 0.060) and histological subtypes (V = 0.109).

**Conclusions:**

More than one-third of the GC patients in our hospital had positive MN-FH. The most frequent forms of MN-FH were esophageal cancer and GC. The prevalence of positive MN-FH was not correlated to any of the clinicopathologic features, including sex, age and histological subtypes in the study population of GC patients.

## Background

Gastric cancer (GC) is one of the most common malignant tumors diagnosed worldwide. Although the incidence of GC in many developed countries has shown a downward trend over the past decade, over 980,000 new cases were estimated in 2008 [1], 70% of which occurred in developing countries. GC is not only the second most common malignant tumor reported in China [1], but accounted for more than 460,000 new cases and more than 350,000 deaths in 2008 alone [2].

The majority of GC cases worldwide are believed to be sporadic. Only about 10% of GC cases show familial aggregation, which is defined as two or more GC patients in close relatives [3]. Furthermore, only 1 to 3% of GC cases have been diagnosed as hereditary syndromes [4]. Familial gastric cancer (FGC) has been associated with environmental factors, such as *Helicobacter pylori* infection and a high salt diet, and genetic factors, such as E-cadherin mutations [5,6]; however, it is possible that interactions between environmental and genetic factors may increase the risk of FGC or promote its pathogenic progress.

Familial history of malignant neoplasm (MN-FH) has been characterized as a risk factor of GC, due to the fact that close family members are often exposed to similar environmental risk factors and are subject to inherited genetic susceptibility [7]. However, the exact environmental and genetic factors differ for families in different areas [8], creating a regional disparity for MN-FH risk. Therefore, determining the prevalence of MN-FH in particular regions and evaluating its relation with the clinicopathologic features of GC patients may help in designing effective diagnostic and therapeutic strategies to reduce the number of cases of GC in high-risk areas, such as in China. Few studies [9-12] to date have evaluated GC-related MN-FH, and their findings have been largely inconsistent. We conducted a study of GC patients in the Shandong Province of in the north of China to determine if MN-FH was a risk factor for GC and whether it was related to any clinicopathologic features of current GC patients.

## Methods

A total of 104 patients diagnosed with primary gastric adenocarcinoma and hospitalized in the Department of Gastrointestinal Surgery at Liaocheng People’s Hospital (Shandong, China) from January 2008 to December 2009, was enrolled in our prospective study. The MN-FH for each case was determined upon admission by a single investigator (JXY), who relied on patient self-reporting or reporting by an accompanying spouse or adult-age child. Positive MN-FH was defined as malignant neoplasm-affected first- and second-degree relatives of the current GC case.

The study did not include any intervention beyond the prescribed treatment regimens and histological types, (defined by World Health Organization (WHO) standards [13]), which were obtained from the patient records. The study was approved by the Ethical Committee of Liaocheng People’s Hospital.

All statistical analyses were carried out with SAS software v9.0 (SAS Institute, Cary, NC, USA). The Chi-square (*χ*^2^) test was used to evaluate the correlation between MN-FH prevalence and various clinicopathological features using the Cramer’s V coefficient. Statistical significance was indicated by a *P*-value less than 0.05.

## Results

Of the 104 GC cases in this study, 76 were male and 28 were female. The median age was 58 years old (range: 22 to 80 years). Thirty-seven (35.6%) of the GC cases had positive MN-FH, with 42 associated tumors in first- and second-degree relatives.

The 42 associated malignant tumors and their frequencies are listed in Table 1. Twenty-six (61.9%) of the associated tumors were located in the digestive system (esophagus, stomach, liver and colon). Twenty-two (52.4%) of the associated tumors were located in the alimentary tract (esophagus, stomach and colon). The ratios of esophageal cancer and gastric cancer MN-FH were 26.2% and 23.8%, respectively. Those cancers very rarely represented by the associated tumors (each 1/42) included colon, breast, brain, nasopharangeal and ocular.

**Table 1:** The location of associated tumors in gastric cancer patients with positive malignant neoplasm family history

**Associated malignant neoplasm**	**Number**	**Constituent ratio, %**
Esophageal carcinoma	11	26.2
Gastric cancer	10	23.8
Liver cancer	4	9.5
Lung cancer	4	9.5
Cervical/endometrial cancer	2	4.8
Colon cancer	1	2.4
Breast cancer	1	2.4
Brain glioma	1	2.4
Nasopharyngeal carcinoma	1	2.4
Eye malignant neoplasm	1	2.4
Uncertain malignant neoplasm	6	14.3
Total	42	100

As shown in Table 2, there were no significant differences in the MN-FH prevalence among different sexes (V = 0.044), ages (V = 0.060), or histological subtypes (V = 0.109) of the current GC patients (all, *P *> 0.05).

**Table 2:** The prevalence of malignant neoplasm family history among different clinicopathologic features of gastric cancer patients

**Clinicopathologic parameter**	**Number of total patients**	**Number of patientswith positive MN-FH, n (%)**	**Cramer’s V**	***χ***^**2**^	***P*-value**
Sex			0.044	0.197	0.657
Male	76	28 (36.8)			
Female	28	9 (32.1)			
Ages, years			0.060	0.368	0.832
<45	22	9 (40.9)			
45 to 60	39	13 (33.3)			
>60	43	15 (34.9)			
Histology					
TUB^a^	19	5 (26.3)	0.109		0.776*
POR^b^	69	27 (39.1)			
SIG^c^	10	3 (30.0)			
MUC^d^	6	2 (33.3)			

*: Fisher's exact test.

^a^TUB, tubular adenocarcinoma; ^b^POR, poorly differentiated adenocarcinoma; ^c^SIG, signet-ring cell carcinoma; ^d^MUC, mucinous adenocarcinoma.

## Discussion

### Family history of malignant neoplasm as a risk factor for GC

An earlier large-scale retrospective study [14] had reported that family history is a sufficiently reliable predictor of cancer risk for all types of cancers. While our study was smaller in scale (n = 104) and more focused (all Chinese from a single region), it was prospective in nature and, unlike the earlier study, not solely based on a questionnaire administered by multiple investigators. The author (JXY) as a single investigator collected data in-person with the aim of increasing our study’s reliability.

Our results showed 35.6% of 104 patients with GC presented with MN-FH in first- and second-degree relatives. This percentage is notably lower than that reported from a study in Japan (46.4%) [11]; however, the higher GC incidence in Japan may account for the overall higher MN-FH prevalence. Another study of Italian GC patients reported MN-FH for 70.8% of the study population [10]. It is possible that this remarkably high MN-FH may reflect an ethnicity-related genetic susceptibility. Ten (9.61%) of the 104 GC patients in our study had one or more first- and second-degree relatives with GC. This familial clustering of GC agrees with previous reports stating that approximately 90% of GC cases are sporadic and only approximately 10% present familial clustering [3,4].

### Most MN-FH associated tumors of GC patients were located in the digestive system, especially in the esophagus and stomach

Our results showed that more than one-half (61.9%) of associated tumors in MN-FH were located in the digestive system. Likewise, a previous study of MN-FH in second-degree relatives of Chinese GC patients from the Guandong Province in the south of China determined that 74.9% of associated tumors were located in the stomach, esophagus, liver and colorectum. A study of MN-FH in Japanese GC patients also found that 70.9% of total associated malignant neoplasms involved organs of the digestive system (stomach, colorectum, liver, esophagus and pancreas) [11]. However, the reported prevalence of each organ-specific MN-FH-associated tumor was different for different regions. Our study population from north of China had the highest amount of associated tumors in the esophagus (26.2%), followed by the stomach (23.8%). The report cited above, using GC patients from south of China, found notably different percentages of stomach- and esophagus-associated tumors (38.6% and 18.3%, respectively) [15]. In contrast, the study of Japanese GC patients found that 40.9% of associated tumors were located in the stomach [11], while the study of Italian GC patients found that only 21.9% of associated tumors were located in the stomach [10]. The exact reasons for this regional disparity should be analyzed in future studies that consider ethnic-related factors, such as heredity, and environmental exposures, such as geographic or cultural factors.

The risk of GC was higher in individuals with a positive MN-FH of digestive cancers. A case–control study from the USA found that the risk for GC patients was increased by an MN-FH of digestive cancers, even after adjusting for other risk factors, such as age, race, smoking and body mass index (BMI) [16]. Another case–control study of GC patients in Taiwan identified positive family history of GC as a significant risk factor for GC [17]. Again, the MN-FH risk determined by each of the studies using different patient populations showed regional differences. A review of the related literature indicated that the risk ratio was higher in Asians than in Europeans [18]. Specifically, the relative risk for GC in individuals with a positive family history of GC varied from 1.5-fold to 3.5-fold, compared with individuals with a negative family history of GC [18]. Thus, it is not surprising that our study group had unique profiles of organ-specific associated tumors from MN-FH.

### Colorectal and breast cancers are not common associated tumors in GC patients

Colorectal cancer has been identified as a common associated tumor in MN-FH by previous studies of non-Chinese GC patient populations. It ranked second among the associated tumors reported by both the Italian study (11.1%) [10] and the Japanese study (16%) [11]. In our study population, however, only one of the 42 MN-FH-associated tumors was colorectal cancer.

Breast cancer also ranked high (third place; 10.2%) among the associated tumors in the Italian study [10]. In contrast, breast cancer ranked low (sixth place) in the Japanese study, which found that only 4.3% of associated tumors were breast cancers [11]. Our result was consistent with the Japanese study, suggesting that some Asian-specific factors may contribute to the MN-FH risk of GC.

It is also possible that these results may simply reflect the different incidences of colorectal cancer and breast cancer in different regions [18]. It is well recognized that incidences of colorectal cancer and breast cancer are higher in Europe than in Japan or China. In addition, European countries have a higher rate of hereditary diffuse gastric cancer (HDGC) than Asian countries. HDGC family history has been associated with breast cancer [19,20], the etiology of which may involve germline mutations in the cadherin-1 (*CDH1*) gene.

### Prevalence of MN-FH does not correlate to GC patient sex, age or histological subtype in Chinese patients

Similar to the Italian study [10], our results showed that there was no correlation between prevalence of MN-FH and sex of GC patients. Specifically, Bernini *et al*. reported that there were no significant differences in sex among patients with and without a family history of GC [10]. The study of GC patients by Lee *et al*. [21] also found no differences among males and females in relation to positive family history of cancer.

As for the putative relation with age, the Italian study found no significant correlations between the GC patients with and without a family history of GC (mean ages of 65.2 and 67.6, respectively) [10], which was similar to our findings. The Japanese study also found no correlation with age (mean ages of patients with family history of GC, with a family history of other cancers, and without a family history of cancer were 64.4, 64.5 and 67.7 years, respectively) [11]. Finally, a comparative analysis of familial and non-familial cancers in USA-based patients found no significant difference in diagnostic age with adenocarcinomas of the esophagus and the gastroesophageal junction [22].

As for the putative relation between GC histological subtypes and MN-FH, both our study and the Japanese study [11] found no significant differences among GC patients. However, an earlier Japanese study [23] did report an increased risk of intestinal type GC when both parents had GC (odds ratio = 7.8), and an increased risk of diffuse-type cancer when both parents had non-GC (odds ratio = 2.1). Intriguingly, the Italian study found that the gastric adenocarcinoma type, as determined by the Lauren classification system, was correlated to family histories of GC [10]. Specifically, the patients with positive family histories of GC were found to present with more intestinal type GC than those with negative family histories of GC (71.8% *vs.* 55.1%, respectively).

## Conclusions

More than one-third of the patients had at least one first- or second-degree relative with a cancer diagnosis. The majority of the associated tumors involved digestive system organs, with the esophagus and stomach being the most frequently represented, suggesting that a family history of digestive system cancers may be a risk factor for some GC patients. Colorectal and breast cancers, which have been previously associated with an MN-FH of GC in different ethnicities, were rare MN-FH types in our study population. The MN-FH prevalence was not correlated with patient sex, age or histological subtype.

## Abbreviations

CDH1: Cadherin-1 gene; BMI: Body mass index; FGC: Familial gastric cancer; GC: Gastric cancer; HDGC: Hereditary diffuse gastric cancer; MN-FH: Malignant neoplasm family history; WHO: World Health Organization.

## Competing interests

The authors declare that they have no competing interests.

## Authors’ contributions

JXY conceived this study, collected data, performed analysis and drafted the manuscript. BF participated in study design and drafted the manuscript. QZ collected data and performed data analysis. All authors read and approved the final manuscript.
